# Calculation of Cement Composition Using a New Model Compared to the Bogue Model

**DOI:** 10.3390/ma14164663

**Published:** 2021-08-19

**Authors:** Sang-Hyo Shim, Tae-Hee Lee, Seong-Joon Yang, Norhazilan Bin Md. Noor, Jang-Ho-Jay Kim

**Affiliations:** 1 POSCO Engineering and Construction, Incheon Tower-Daero, Yeonsu-gu, Incheon 22009, Korea; ssh7772@naver.com; 2 School of Civil and Environmental Engineering, Yonsei University, Seoul 03722, Korea; saintlth@yonsei.ac.kr (T.-H.L.); sjyang933@yonsei.ac.kr (S.-J.Y.); 3 Department of Structure and Materials, Universiti Teknologi Malaysia, Skudai 81310, Malaysia; norhazilan@utm.my

**Keywords:** cement compound, composition ratio, Bogue model, chemical composition

## Abstract

The major cement composition ratios of alite, belite, aluminate, and ferrite have been calculated with the Bogue models until now. However, a recent comprehensive analysis based on various experimental data has revealed that the chemical composition of alite, belite, aluminate, and ferrite implemented by the Bogue models are slightly different than the experimental data, where small amounts of Al_2_O_3_ and Fe_2_O_3_ existing in alite and belite can change the prediction of cement composition. Since the amounts of cement compound are very important factors in determining the properties of concrete, improvement in the calculation would give more precise prediction for application usages such as climate change adaptable cement and high durable concrete manufacturing. For this purpose, 20 new models are proposed by modifying chemical compositions of the cement compounds and verified with the 50 experimental data sets. From the verification, the most accurate models are identified. The calculation using new models exhibit an accuracy improvement of approximately 5% compared to the Bogue models. Their applicable range is also presented. The study results are discussed in detail in the paper.

## 1. Introduction

Cement is produced by heating limestone and clay at approximately 1500 °C in a kiln, which induces the chemical reactions that produce cement compounds. Mindess [1] suggested that the main compounds are alite, belite, aluminate, and ferrite, which constitute approximately 90% of the total cement weight. Cement is generally classified into five types, by ASTM C150 [2], based on the compressive strength generation time and hydration heat output. Table 1 lists the physical specifications for each type. Their different properties result from the different composition ratios of their compounds, which have different hydration characteristics. For example, cement types 2 and 4 have a high belite content, which leads to low initial hydration heat and high long-term strength; type 3 has a high aluminate ratio, corresponding to high initial strength. Table 2 summarizes the composition of the cement types, which is an important parameter because it ultimately controls the concretes’ properties. The cement compounds are generally quantified by using the Bogue models proposed in 1955 [3,4]. However, a recent comprehensive analysis of these models based on various experimental data has shown some shortcomings in their prediction accuracy; in particular, the calculated alite and belite content percentages were 5–10% lower and higher, respectively, than the experimental data [5].

**Figure 1:**
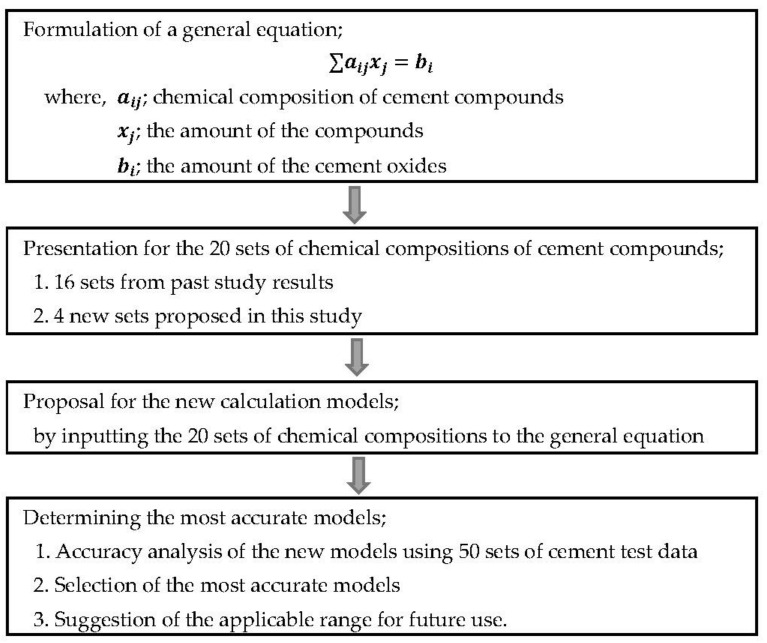
Overall procedure for the development of more accurate models to predict the cement composition.

Kristmann [6] suggested that alite calculated by the Bogue model is 8.3% less than experimental data and belite is 6.0% more. Taylor [7] pointed out alite calculated by the Bogue model is higher than experimental data in many studies. Stutzman [8] suggested that the uncertainty in the Bogue model is 1.4–9.6%. Diana [9] reported C_3_A amounts by Bogue are 0.6% more than Rietveld’s experimental value. Aldrige [10] presented the comparison of the experimental results using a microscope and X-rays for six cement samples with the calculation results using the Bogue model. As a result, the Bogue model was calculated to be 7.1% less for alite, 1.0% less for belite, and 0.1% and 0.7% more for aluminate and ferrite, respectively. Sayed Horkoss [11] presented C_3_A percentages calculated by the Bogue model in 12 high sulfur clinkers were 2.0% more than experimental data and proposed a new model for C_3_A. Islem Labide [12] compared the value calculated by the Bogue model with the Rietveld experimental value and suggested that the amount of C_3_S + C_2_S by experiment is higher than the Bogue model and the amount of C_3_A+C_4_AF is lower. Stutzman [13] suggested the reliability of the Bogue model using statistical techniques, and Bezerra [14] suggested the amount of oilwell cement compound through the Bogue model, Taylor model, and optical microscopy experiments. Many studies point out the chemical composition of cement compounds as the main reason for the difference between the Bogue model and the experimental values [8,15,16,17]. The Bogue model is derived by defining the chemical composition of cement compounds as C_3_S for alite, C_2_S for belite, C_3_A for aluminate, and C_4_AF for ferrite [18]. However, Taylor [19] suggested that there are several micro-compounds in the cement compound, and Harrison [20] also presented similar results through 111 sample experiments. Since the amounts of cement compounds are very important to accurately determine the concrete properties, the accuracy of the Bogue models must be verified experimentally. Therefore, 50 experimental data sets were collected and compared to the calculated results using the Bogue models. The verification revealed errors in the calculations and, thus, the need for improving these models. In the present study, this improvement was achieved by selecting the most accurate prediction models among newly proposed ones. Additionally, based on the result analysis, the applicable ranges of the new models are proposed and discussed. Figure 1 displays the overall flowchart of this study to clarify the process of proposal, identification, and verification of the new prediction models.

## 2. Evaluation of the Bogue Model

### 2.1. Bogue Model

Bogue proposed a method in 1929 and some models in 1955 to quantify the composition of cement compounds [3,4]. To develop the models including the CaO, SiO_2_, Al_2_O_3_, and Fe_2_O_3_ terms denoted with C, S, A, and F, respectively, the following assumptions are used.

Fe_2_O_3_ reacts with Al_2_O_3_ and CaO to form C_4_AF.
The remaining Al_2_O_3_ reacts with CaO to yield C_3_A.
The remaining CaO reacts with SiO_2_, forming C_2_S that successively reacts with any CaO left over to give C_3_S. After these reactions, eventually, the unreacted CaO remains uncombined.
MgO remains essentially uncombined.

Based on these assumptions, the produced cement compounds are C_3_S, C_2_S, C_3_A, and C_4_AF. When their chemical compositions are defined, the models to calculate their amounts according to the initial raw oxides of CaO, SiO_2_, Al_2_O_3_, and Fe_2_O_3_ can be derived as follows [4]:

C_3_S% = (4.0710 × CaO%) − (7.6024 × SiO_2_%) − (6.7187 × Al_2_O_3_%) − (1.4297 × Fe_2_O_3_%) (1)

C_2_S% = − (3.0710 × CaO%) + (8.6024 × SiO_2_%) + (5.0683 × Al_2_O_3_%) + (1.0785 × Fe_2_O_3_%) = (2.8675 × SiO_2_%) − (0.7544 × C_3_S%) (2)

C_3_A% = (2.6504 × Al_2_O_3_%) − (1.6920 × Fe_2_O_3_%) (3)

C_4_AF% = (3.0432 × Fe_2_O_3_%) (4)

In commercially produced cement, gypsum is also added to delay the natural hardening. Since the main component of gypsum is CaO·SO_3_, the models shall be modified to consider its composition as well. ASTM C150 defined the following models for this purpose [2]:

C_3_S% = (4.071 × CaO%) − (7.600 × SiO_2_%) − (6.718 × Al_2_O_3_%) − (1.430 × Fe_2_O_3_%) − (2.852 × SO_3_%) − (5.188 × CO_2_%) (5)

C_2_S% = (2.867 × SiO_2_%) − (0.7544 × C_3_S%) (6)

C_3_A% = (2.650 × Al_2_O_3_%) − (1.692 × Fe_2_O_3_%) (7)

C_4_AF% = (3.043 × Fe_2_O_3_%) (8)

The above models are valid only when A/F < 0.64; in other cases, they must be further modified since the type of cement compounds produced are different.

### 2.2. Experimental Data

To evaluate the accuracy of the Bogue models, 50 experimental data sets were collected from past studies. Kristmann estimated the amount of the oxides and compounds in 39 commercial types of cement through microscopic examination and X-ray diffraction [15]. Le Saoût obtained such information for five cement types and one clinker via energy-dispersive spectroscopy [16]. Jadhav reported the oxide and compound contents for three cement types [21]. Scrivenera derived the amount of the oxides and the compounds for one cement type from X-ray diffractometry experiments [22]. Paweł measured the amounts of oxides and compounds via, respectively, X-ray fluorescence spectroscopy and XRD experiments [17]. All these data are presented in Appendix A.

### 2.3. Accuracy of the Bogue Models

**Figure 2:**
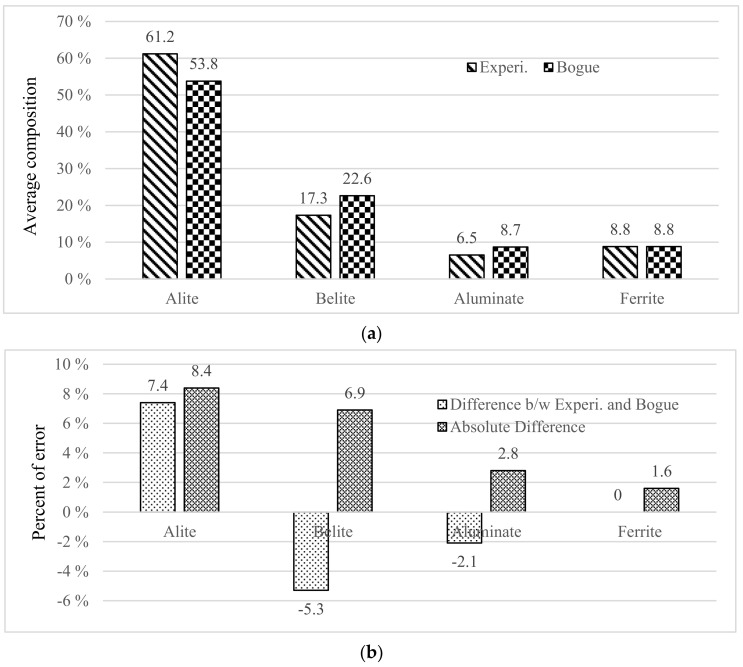
Comparison of experimental and calculated (by the Bogue models) cement compound contents: (**a**) the average values of experimental and calculated cement compound contents; (**b**) the average differences and the average absolute differences between the two, where the differences are percentages of error from experiment and Bogue model.

To evaluate the accuracy of the Bogue models, the cement compound contents were calculated using the equations based on the amounts of the five raw oxides (i.e., CaO, SiO_2_, Al_2_O_3_, Fe_2_O_3_, and SO_3_). Since the oxide amounts were obtained experimentally (Appendix A), their sum was not 100%. Therefore, in this study, the sum was adjusted to 100% for the calculation input. Figure 2 compares the experimental data about the cement compound contents with the as-calculated results by showing their average values and the differences between them. As shown in Figure 2b, the average calculated alite and belite contents were 7.4% lower and 5.3% higher, respectively, than the experimental ones, with corresponding average absolute differences of 8.4% and 6.9%, respectively. The average absolute differences were higher than the average ones because these positive and negative differences between experimental and calculated values did not offset each other; therefore, the average absolute difference could allow a more accurate comparison and was used as an indicator to evaluate the accuracy. In general, the value calculated via the Bogue models tends to underestimate and overestimate alite and belite by 5–10%, respectively, compared to the experimental data. As described above, a similar tendency was observed also in this study.

Alite and belite differ in the hydration reaction rate and hydration heat generated. Mindess suggested the following relationship between the cement hydration heat and the cement compound contents [1]:

H3days (kJ/kg) = (240 × C_3_S) + (50 × C_2_S) + (880 × C_3_A) + (290 × C_4_AF) (9)

H1year (kJ/kg) = (490 × C_3_S) + (225 × C_2_S) + (1160 × C_3_A) + (375 × C_4_AF) (10)

where H3days is the hydration heat of unit cement amount after 3 days of curing and H1year is that after 1 year; the unit of constants is kJ/kg.

With the above models, the concrete hydration heat was calculated according to the change in the cement compound amounts as shown in Table 3, where the mass of concrete is assumed to be 2350 kg and the specific heat 1 kJ/kg°C. In the case of concrete made with 400 kg of cement with 7% less alite and 7% more belite, the as-calculated hydration heat was 2.2 °C (5.1%) lower in 3 days of curing. Therefore, when predicting the concrete characteristics based on the Bogue models, the calculated initial hydration heat may underestimate the real value, which means that temperature cracking may occur earlier than expected. Hence, the cement compound contents should be calculated more accurately.

## 3. Definition and Validation of the General Equation for Cement Composition Calculation

### 3.1. Definition of General Equation

A general equation and a compositional model are needed to develop new calculation for the cement compounds. In this section, the general equation is first proposed, and the validation of the equation will be verified using the Bogue and the Taylor model. Since the chemical compositions of cement oxides are defined and fixed, their molecular weights are constant. If the chemical compositions of the cement compounds produced are known, their amounts could be derived from the molecular weights of these oxides. Such a calculation method can be modeled as follows. By constructing a linear equation for the oxide compositions in each cement compound and finding the solution, the compound amounts can be expressed as

(11) $$\sum a_{ij}x_{j}=b_{i}$$

where $x_{j}$ is the amounts of the cement compound; $b_{i}$ is the amounts of the oxides, which is generally provided by the cement manufacturers; $a_{ij}$ is the oxide ratio of the cement compound or chemical composition. Thus, if the chemical compositions of the cement compounds are known, their amounts can be calculated by obtaining the inverse of Equation (11).

### 3.2. Validation by Using the Bogue Models

To derive the Bogue models from the general equations presented in Section 3.1, the chemical composition of the cement compounds assumed by Bogue must be substituted in these general equations.

Weight ratios in Table 4 are substituted to the $a_{ij}$ in Equation (11) and whose inverse matrix is

(12) $$\left[\begin{array}{c} C_{3}S\\ C_{2}S\\ C_{3}A\\ C_{4}\mathrm{AF} \end{array}\right]\;=\left[\begin{array}{c} 4.071\\ -3.072\\ -\\ - \end{array}\begin{array}{c} -7.600\\ \;\;\;\;8.600\;\;\;\;\;\\ -\\ - \end{array}\begin{array}{c} -6.718\;\;\\ \;\;5.068\;\;\;\;\;\\ 2.650\;\;\;\\ - \end{array}\begin{array}{c} -1.430\\ 1.079\\ -1.692\\ 3.043 \end{array}\right]\left[\begin{array}{c} \mathrm{CaO}\\ {\mathrm{SiO}}_{2}\\ {\mathrm{Al}}_{2}O_{3}\\ {\mathrm{Fe}}_{2}O_{3} \end{array}\right]$$

Since these models are similar to those presented in the Bogue model, the general equations proposed in this study can be considered valid.

### 3.3. Validation by Using the Taylor Models

Taylor suggested the chemical compositions of cement compounds as weight ratios of oxides as shown in Table 5 [7]. However, the as-defined chemical compositions are applicable only when 1.65% MgO and 2.8% Fe_2_O_3_ are contained in the cement; if these values change, the chemical compositions should also vary. The values represented by the molecular quantities in Table 5 are the same as the CaO quantities assumed in the Bogue models. In addition, the molecular quantities of the remaining oxides are calculated based on this assumption. In the Bogue and Taylor models, the SiO_2_ content in alite is assumed to be 1 and 0.985, respectively; this difference is due to the molecular quantities of the remaining oxides. Taylor defined the following models [7]:

Alite% = (4.641200 × CaO%) − (8.838681 × SiO_2_%) − (7.094597 × Al_2_O_3_%) − (1.544488 × Fe_2_O_3_%) (13)

Belite% = (−3.724144 × CaO%) + (10.29531 × SiO_2_%) + (5.343733 × Al_2_O_3_%) + (1.065700 × Fe_2_O_3_%) (14)

Aluminate% = (0.117872 × CaO%) − (0.369269 × SiO_2_%) + (3.669829 × Al_2_O_3_%) − (3.955085 × Fe_2_O_3_%) (15)

Ferrite% = (−0.023283 × CaO%) − (0.055861 × SiO2%) − (0.867256 × Al2O3%) + (5.621492 × Fe2O3%)(16)

These models can be expressed in a matrix form by inserting chemical compositions given in Table 5 into the $a_{ij}$ in the general equations, and its inverse matrix can be calculated. Taylor stated that the chemical composition of the cement compounds varies depending on the MgO and Fe_2_O_3_ amounts. Therefore, if the oxide contents change, the models must also change.

### 3.4. Modification of the Taylor Models by Considering the Sulfate Component

The Taylor models do not consider gypsum when calculating the amount of the cement compounds; Taylor discussed the calculation method for the weight of the sulfate component (i.e., K_2_SO_4_, Na_2_SO_4_, and CaSO_4_) separately. However, CaSO_4_ should be considered in such models. Since the amount of SO_3_ contained in the cement increases, the CaO amount consumed by CaSO_4_ also increases. Therefore, this phenomenon must be taken into account. The calculations considering the sulfate components can be obtained as follows. First, the sulfate components are added to the chemical compositions, obtaining the following matrix:

(17) $$\left[\begin{array}{c} \mathrm{CaO}\\ {\mathrm{SiO}}_{2}\\ {\mathrm{Al}}_{2}O_{3}\\ {\mathrm{Fe}}_{2}O_{3}\\ {\mathrm{SO}}_{3}\\ K_{2}O\\ {\mathrm{Na}}_{2}O \end{array}\right]=\left[\begin{array}{ccccccc} 0.716 & 0.635 & 0.566 & 0.475 & 0.412 & - & -\\ 0.252 & 0.315 & 0.037 & 0.036 & - & - & -\\ 0.010 & 0.021 & 0.313 & 0.219 & - & - & -\\ 0.007 & 0.009 & 0.051 & 0.214 & - & - & -\\ - & 0.001 & - & - & 0.588 & 0.459 & 0.564\\ 0.001 & 0.009 & 0.007 & 0.002 & - & 0.541 & -\\ 0.001 & 0.001 & 0.010 & 0.001 & - & - & 0.436 \end{array}\right]\left[\begin{array}{c} \mathrm{Alite}\\ \mathrm{Belite}\\ \mathrm{Aluminate}\\ \mathrm{Ferrite}\\ \mathrm{Ca}{\mathrm{SO}}_{4}\\ K_{2}{\mathrm{SO}}_{4}\\ {\mathrm{Na}}_{2}{\mathrm{SO}}_{4} \end{array}\right]$$

Then, with the inverse of this matrix, the following modified Taylor models are derived:

(18) $$\left[\begin{array}{c} \mathrm{Alite}\\ \mathrm{Belite}\\ \mathrm{Aluminate}\\ \mathrm{Ferrite}\\ \mathrm{Ca}{\mathrm{SO}}_{4}\\ K_{2}{\mathrm{SO}}_{4}\\ {\mathrm{Na}}_{2}{\mathrm{SO}}_{4} \end{array}\right]=\left[\begin{array}{ccccccc} 4.634 & -8.897 & -7.301 & -1.362 & -3.245 & 2.758 & 4.192\\ -3.718 & 10.342 & 5.509 & 0.911 & 2.604 & -2.213 & -3.364\\ 0.118 & -0.371 & 3.665 & -3.950 & -0.082 & 0.070 & 0.106\\ -0.023 & -0.056 & -0.886 & 5.621 & 0.016 & -0.014 & -0.021\\ -0.030 & 0.095 & 0.161 & -0.091 & 1.721 & -1.463 & -2.223\\ 0.052 & -0.151 & -0.122 & 0.018 & -0.036 & 1.881 & 0.047\\ -0.005 & 0.005 & -0.078 & 0.078 & 0.003 & -0.003 & 2.287 \end{array}\right]\left[\begin{array}{c} \mathrm{CaO}\\ {\mathrm{SiO}}_{2}\\ {\mathrm{Al}}_{2}O_{3}\\ {\mathrm{Fe}}_{2}O_{3}\\ {\mathrm{SO}}_{3}\\ K_{2}O\\ {\mathrm{Na}}_{2}O \end{array}\right]$$

## 4. Proposal of New Models to Calculate the Cement Compound Contents

### 4.1. New Chemical Compositions of the Cement Compounds

The new chemical compositions of the four main cement compounds were defined based on the 16 chemical compositions collected from previous studies. Kristmann experimentally estimated the compositions of 28 types of alite and belite and four types of aluminate and ferrite; the corresponding values are summarized in Table 6 [15]. Stutzman reported the chemical compositions of these four compounds for 14 cement types (Table 7), and 8 of these compositions were used for the present study [8]. Le Saoût identified the chemical compositions of the cement compounds as molar ratios through EDS experiments on five cement types and one clinker, and three of these compositions were used in the present study; the values in Table 8 were obtained by converting the molecular quantities into weight ratios [16]. Paweł also presented the chemical compositions of cement compounds as weight ratios as shown in Table 8 [17]. The chemical compositions listed in Table 8 indicate that all the cement compounds contain CaO, SiO_2_, Al_2_O_3_, and Fe_2_O_3_, unlike for the assumptions of the Bogue models. For example, in the cases of alite and belite, only CaO and SiO_2_ are considered in the Bogue models, while also small amounts of Al_2_O_3_ and Fe_2_O_3_ and even SO_3_ are present in the chemical compositions determined by Paweł, which is consistent with those of the Taylor models. In this paper, in addition to the chemical composition collected from past studies, the following four chemical compositions are also proposed as shown in Table 9.

- Case Average: average chemical compositions of 16 cases from past studies.
- Case 1: chemical compositions based on the Bogue models + minor oxides of all the cement compounds, not considered in the Bogue models.
- Case 2: chemical compositions based on the Bogue models + minor oxides of alite and belite, not considered in the Bogue models.
- Case 3: chemical compositions based on the Bogue models + minor oxides of aluminate and ferrite, not considered in the Bogue models.

where, in Cases 1–3, the amounts of the minor oxides are derived from the chemical composition of Case Average.

### 4.2. New Models

New models were defined by inputting the chemical compositions presented in Section 4.1 into the general equations (i.e., Equation (11)). The chemical compositions given in Section 4 correspond to the $a_{ij}$ term of the Equation (11), but the sum of $a_{nj}$ is not 100%, since the data were experimentally obtained. Since the sums of the inputs and outputs differ when using raw data for the analysis, data calibrations were required; thus, the sum of $a_{nj}$ was adjusted to 100% by dividing the data of each cement composition by the sum of all compositions of each compound and used to construct the matrix, from which the inverse matrix was derived (i.e., the models for calculating the cement compound amounts). The new $a_{ij}$s and calculation models are given in Appendix B. By inputting the cement oxides shown in Appendix A into the proposed models, the amount of the cement compounds can be calculated.

### 4.3. Accuracy Analysis of the New Models

**Figure 3:**
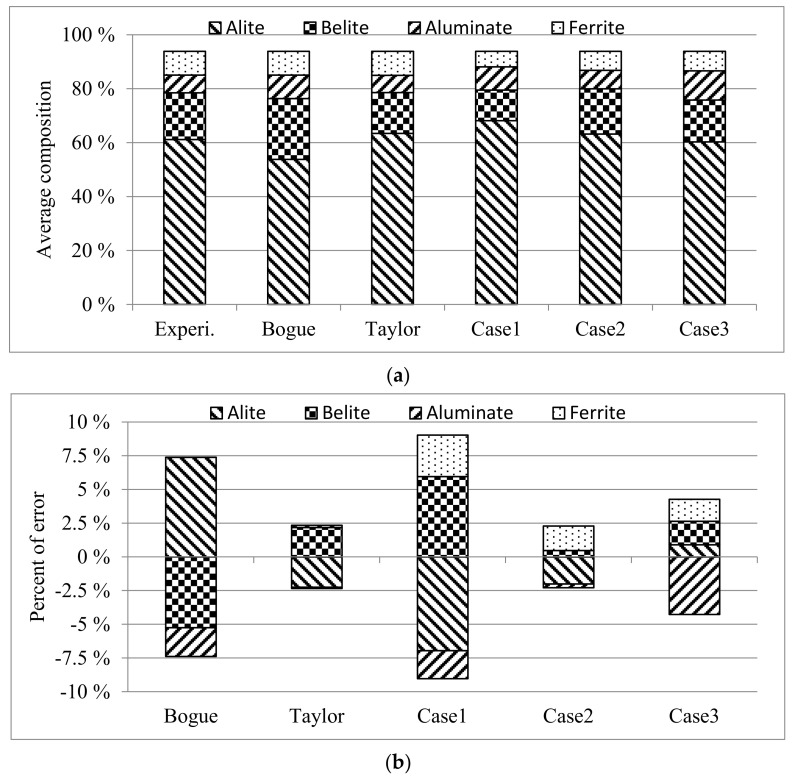

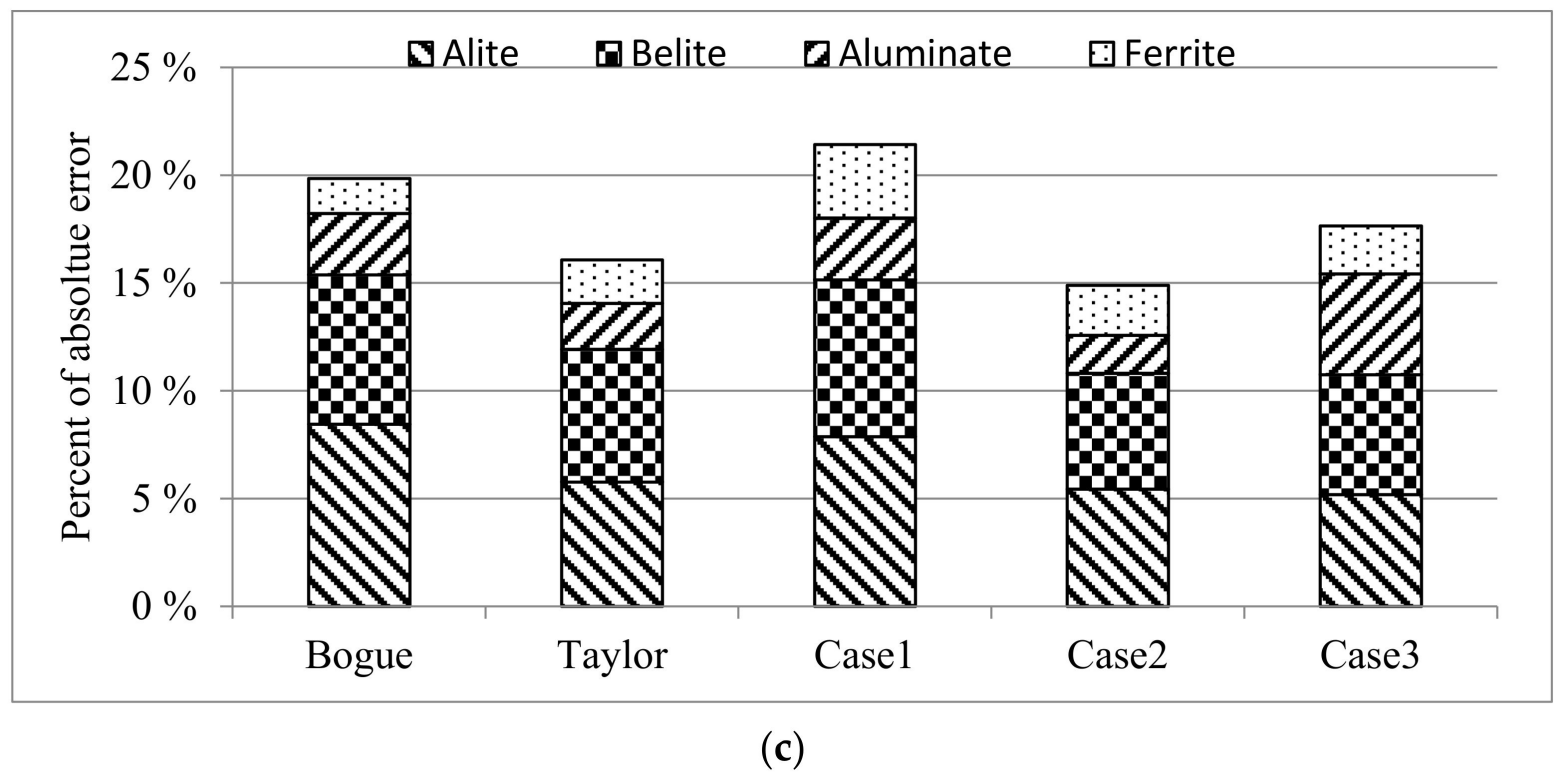
Results obtained experimentally and with the Bogue, Taylor, and Case 1–3 models: (**a**) average values of 50 experimental data sets and calculated results; (**b**) the average differences between experimental data sets and calculated results; (**c**) the average absolute differences between experimental data sets and calculated results.

**Figure 4:**
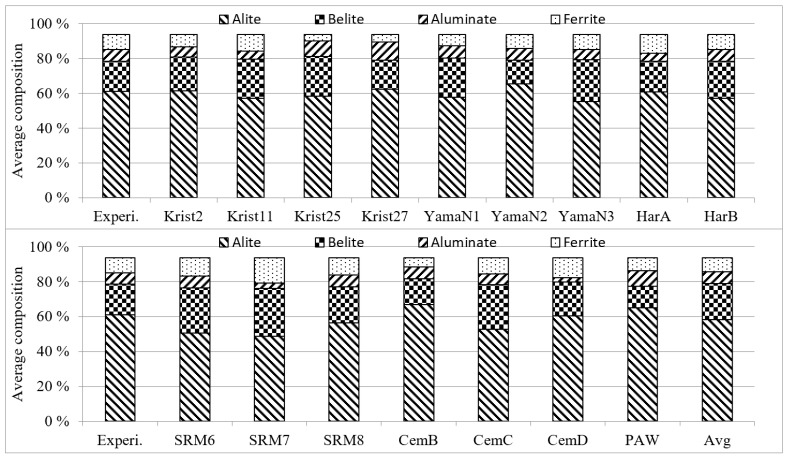
Experimental data and results of the 17 model sets given in Appendix B.

To evaluate the accuracy of the new models, the oxide amounts presented in Appendix A were put into the models given in Appendix B for calculating cement compound contents and compared with the experimental data. The oxide amounts were obtained from the chemical experiments, and thus, their sum was not 100%; hence, the sum was adjusted to 100% for the calculation input. The weights of the five oxides (i.e., CaO, SiO_2_, Al_2_O_3_, Fe_2_O_3_, and SO_3_) were used for the calculations. Figure 3 compares the results from the three models using the chemical compositions of Cases 1–3 with those obtained by the Bogue and Taylor models. The compared compounds are alite, belite, aluminate, and ferrite, presented in Appendix A. For consistency, the sum of these four compounds was adjusted to be equal to that obtained with the Bogue models. As shown in Figure 3b,c, Cases 2 and 3 exhibited smaller values than the Bogue results. The comparison also demonstrates that the results calculated via the Taylor models are highly accurate, but Case 2 is the most accurate. Table 10 illustrates the differences between the experimental data and calculated results, as well as the accuracy improvement with respect to the Bogue models. Figure 4 displays the average experimental and calculated values by using the 17 models given in Appendix B. Table 11 lists the average absolute differences between them, as well as the accuracy improvement compared to the Bogue models, which was 2.81% and 2.88% when using the chemical compositions of HarA and SRM8 sets, respectively. However, the accuracy improvement of Case 2 in Table 10 is the highest.

### 4.4. Selection of the Most Accrate Models

Compared to the experimental data, the Case 2 models showed the highest accuracy. Therefore, they were identified as the most accurate new models for cement compound calculation, which can be written as follows.

Alite% = (4.088 × CaO%) − (7.212 × SiO_2_%) − (6.745 × Al_2_O_3_%) − (1.436 × Fe_2_O_3_%) − (2.863 × SO_3_%) (19)

Belite% = − (3.113 × CaO%) + (8.442 × SiO_2_%) + (5.136 × Al_2_O_3_%) + (1.093 × Fe_2_O_3_%) + (2.180 × SO_3_%) (20)

Aluminate% = (0.028 × CaO%) − (0.153 × SiO_2_%) + (2.604 × Al_2_O_3_%) − (1.702 × Fe_2_O_3_%) − (0.020 × SO_3_%) (21)

Ferrite% = − (0.010 × CaO%) − (0.058 × SiO_2_%) + (0.016 × Al_2_O_3_%) + (3.047 × Fe_2_O_3_%) + (0.007 × SO_3_%) (22)

Gypsum% = (0.006 × CaO%) − (0.020 × SiO_2_%) − (0.011 × Al_2_O_3_%) − (0.002 × Fe_2_O_3_%) + (1.696 × SO_3_%) (23)

The error percentage of the new and old models is shown in Table 12, where the unit is a percentage and the values are averages of 50 data.

The cement compound amounts calculated with these models vary depending on the cement oxide contents inputted. An error such as getting a negative value may result from the calculations; to prevent this type of error, the applicable range of these models must be approximately limited. In the case of the Bogue models, two models are proposed according to the A/F value [4]. In a similar manner, the applicable range of the proposed models is limited as follows. If the C/S value of the inputted cement oxides is smaller than the minimum value in Table 13, the alite amount predicted by the proposed model could be negative; if it is greater than the maximum value specified in the table, the calculated belite amount could be negative. If the A/F value is smaller than the minimum value, the calculated aluminate content could be negative. The uncertainties in the experimental measurements of oxide amounts and the compositional variation in cement could potentially affect the accuracy of prediction. Those are studied by Stutzman [8]. He found that the error between the Bogue calculation and the experimental data was 0.06–4.0%, and it increased to 1.4–9.6% if the uncertainties of the input data were included. Further research is needed to understand the uncertainty of the presented model in this paper.

## 5. Conclusions

Alite, belite, aluminate, and ferrite are the main cement compounds and the basic materials of cement hydration. The prediction of their amounts is very important because their hydration heat evolution rate and the final concrete strength mainly depend on their contents. The cement compound amounts are generally calculated with the Bogue models, which were proposed in 1955.

In this study, the accuracy of the Bogue models was verified by comparing the calculated results and experimental data for 50 cases; the results showed that the average alite and belite amounts calculated by the Bogue models were 8.4% lower and 6.9% higher, respectively, than the experimental values, indicating the need for improvement of the models. Therefore, by modifying the chemical composition of the cement compounds, new models were proposed as per the following procedure.

1. General equations for calculating the cement compound amounts were formulated and verified by using the Bogue and Taylor models.
2. To modify the chemical compositions of the cement compounds, 16 compositions were collected from previous studies and 4 new ones were defined. By substituting these compositions into the general equations, new models were obtained.
3. The cement oxides data for 50 cement types were inputted into the proposed models to calculate the amount of the cement compounds, which were then compared with the experimental data to identify the models with the highest accuracy.

Based on the accuracy analysis, the models using the Case 2 chemical compositions that are equivalent to the Bogue chemical compositions with an addition of the minor oxides in alite and belite, not considered in the Bogue models, were selected. Since the average absolute differences of the proposed models improved the accuracy by 4.95% compared to the Bogue models, the cement compound amounts were predicted more accurately with the newly proposed models than with the Bogue models. In the new model, a new term of SO_3_ is added, which was not considered in the Bogue model. SO_3_ is an oxide used in commercial OPC to improve the workability of ready-mixed concrete and other performance improvements. Since the amount of other cement compounds in the model change according to SO_3_ amount, it is significant to recalculate the amount of compounds considered in the model despite the complexity of the new model and relatively small change of approximately 5% in the accuracy of the calculation. Finally, the applicable range of the proposed models was suggested for future usage.

## Figures and Tables

**Table 1:** Standard physical specifications of the cement types as defined in ASTM C150 [2].

Cement Type	Type 1	Type 2	Type 3	Type 4	Type 5
Compressive strength (MPa)					
1 day min.	-	-	12.0	-	-
3 days min.	12.0	10.0	24.0	-	8.0
7 days min.	19.0	17.0	-	7.0	15.0
28 days min.	28.0	28.0	-	17.0	21.0
Heat of hydration (cal/g)					
7 days max	-	70	-	60	-
28 days max	-	-	-	70	-

**Table 2:** Standard composition ratios of the cement types as defined in ASTM C150 [2].

Cement Type	Type 1	Type 2	Type 3	Type 4	Type 5
Tricalcium Silicate(C_3_S) max %	-	-	-	35	-
Dicalcium Silicate(C_2_S) min %	-	-	-	40	-
Tricalcium Aluminate(C_3_A) max %	-	8	15	7	5

where C is an abbreviation of CaO, S is SiO_2_, A is Al_2_O_3_.

**Table 3:** Concrete hydration heat as a function of the change in the cement compound contents.

Case	Compounds Amounts of Cement (%)				Hydration Heat of Cement (kJ/kg)		Hydration Heat of Concrete (°C)	
	C_3_S	C_2_S	C_3_A	C_4_AF	H3 Days	H1 Year	3 Day	1 Year
Case1	55	18	10	8	252.5	456	42.9	77.6
Case2	48	25	10	8	238.9	437.5	40.7	74.5

**Table 4:** Chemical compositions of cement compounds derived from the Bogue models [18].

	Molecular Quantities				Weight Ratio (%)			
Oxide	CaO	SiO_2_	Al_2_O_3_	Fe_2_O_3_	CaO	SiO_2_	Al_2_O_3_	Fe_2_O_3_
Alite	3	1			73.7	26.3	-	-
Belite	2	1			65.1	34.9	-	-
Aluminate	3		1		62.3	-	37.7	-
Ferrite	4		1	1	46.2	-	21.0	32.9

**Table 5:** Chemical compositions of the cement compounds used in the Taylor models [19].

**Weight Ratio (%)**											
**Oxide**	**CaO**	**SiO_2_**	**Al_2_O_3_**	**Fe_2_O_3_**	**MgO**	**SO_3_**	**Na_2_O**	**P_2_O_5_**	**K_2_O**	**TiO_2_**	**Mn_2_O_3_**
Alite	71.6	25.2	1.0	0.7	1.1	-	0.1	0.2	0.1	-	-
Belite	63.5	31.5	2.1	0.9	0.5	0.1	0.1	0.2	0.9	0.2	-
Aluminate	56.6	3.7	31.3	5.1	1.4	-	1.0	-	0.7	0.2	-
Ferrite	47.5	3.6	21.9	21.4	3.0	-	0.1	-	0.2	1.6	0.7
**Molecular Quantities**											
**Oxide**	**CaO**	**SiO_2_**	**Al_2_O_3_**	**Fe_2_O_3_**	**MgO**	**SO_3_**	**Na_2_O**	**P_2_O_5_**	**K_2_O**	**TiO_2_**	**Mn_2_O_3_**
Alite	3.000	0.985	0.023	0.01	0.064	-	0.004	0.003	0.002	-	-
Belite	2.000	0.926	0.036	0.01	0.022	0.002	0.003	0.002	0.017	0.004	-
Aluminate	3.000	0.183	0.912	0.095	0.103	-	0.048	-	0.022	0.007	-
Ferrite	4.000	0.283	1.014	0.633	0.351	-	0.008	-	0.01	0.095	0.021

**Table 6:** Chemical compositions of the cement compounds reported by Kristmann [15].

	**Alite (%)**				**Belite (%)**			
**Case**	**2**	**11**	**25**	**27**	**2**	**11**	**25**	**27**
SiO_2_	25.0	25.3	25.1	25.1	30.5	31.5	31.1	31.7
Al_2_O_3_	1.5	1.4	1.2	1.0	2.8	2.5	2.7	1.5
Fe_2_O_3_	0.9	0.6	1.7	1.0	1.4	1.0	1.5	1.1
CaO	71.0	72.0	72.4	70.2	64.4	64.9	66.4	63.7
MgO	0.8	0.7	0.5	1.2	0.5	0.4	0.3	0.4
Na_2_O	0.1	0.1	0.1	0.1	1.0	0.4	0.4	0.3
K_2_O	0.1	0.1	0.2	0.3	0.2	0.2	0.5	0.5
	**Aluminate (%)**				**Ferrite (%)**			
**Case**	**2**	**11**	**25**	**27**	**2**	**11**	**25**	**27**
SiO_2_	3.7	3.1	4.3	4.3	3.1	2.6	1.8	2.5
Al_2_O_3_	31.0	31.7	31.8	28.7	24.4	22.3	23.0	20.2
Fe_2_O_3_	7.8	5.2	6.3	8.3	22.1	20.8	26.7	25.0
CaO	55.3	58.3	53.3	51.5	49.0	49.9	50.0	47.4
MgO	1.2	1.0	1.8	1.5	2.7	2.7	1.9	3.2
Na_2_O	0.3	0.5	2.6	4.6	0.1	0.1	0.2	0.3
K_2_O	0.6	0.2	0.8	0.2	0.1	0.1	0.2	0.1

**Table 7:** Chemical compositions of the cement compounds reported by Stutzman [8].

Case	YamaN1	YamaN2	YamaN3	Har A	Har B	SRM6	SRM7	SRM8
	Alite (%)							
SiO_2_	24.15	25.13	24.10	25.80	24.60	25.10	24.60	25.70
Al_2_O_3_	1.30	1.17	1.20	1.00	1.20	0.70	1.10	0.50
Fe_2_O_3_	0.66	0.61	0.61	0.40	0.60	0.00	0.00	0.00
CaO	72.76	71.46	72.74	72.60	70.60	72.60	73.40	73.00
SO_3_	0.00	0.00	0.05	0.00	0.00	0.00	0.00	0.30
	**Belite (%)**							
SiO_2_	31.85	33.17	31.62	31.80	31.00	31.80	32.50	33.00
Al_2_O_3_	2.68	1.61	1.99	2.10	2.00	1.00	0.90	1.10
Fe_2_O_3_	1.25	1.05	0.78	0.80	0.90	1.00	0.00	0.00
CaO	62.53	62.38	63.86	63.20	62.90	64.60	65.40	64.70
SO_3_	0.27	0.05	0.26	0.20	0.70	0.20	0.20	0.60
	**Aluminate (%)**							
SiO_2_	4.60	7.10	5.80	4.20	5.00	4.30	4.50	2.40
Al_2_O_3_	27.20	27.50	28.70	31.30	28.10	31.70	28.60	34.90
Fe_2_O_3_	11.40	6.00	5.30	5.00	5.50	3.60	7.50	5.80
CaO	53.00	53.40	54.80	56.00	54.80	57.70	57.30	56.80
SO_3_	0.00	0.00	0.00	0.00	0.00	0.00	0.00	0.00
	**Ferrite (%)**							
SiO_2_	4.30	3.00	4.30	3.80	4.00	4.10	5.00	3.10
Al_2_O_3_	25.10	24.60	24.30	22.10	20.40	20.40	22.10	21.80
Fe_2_O_3_	20.00	22.20	22.10	19.60	20.50	21.60	17.10	24.80
CaO	45.50	44.90	44.50	47.40	47.90	49.20	50.10	48.90
SO_3_	0.00	0.00	0.00	0.10	0.20	0.10	0.10	0.00

**Table 8:** Chemical compositions of the cement compounds reported by Le Saoût and Paweł [16,17].

Case	CemB	CemC	CemD	Paw	CemB	CemC	CemD	Paw
	Alite (%)				Belite (%)			
SiO_2_	24.71	24.45	24.55	25.70	32.09	32.28	32.00	32.85
Al_2_O_3_	2.03	1.12	1.79	1.21	2.07	1.50	2.09	1.74
Fe_2_O_3_	1.41	1.40	1.05	0.56	1.39	0.00	0.93	0.50
CaO	70.42	72.15	71.19	70.38	64.45	66.21	64.98	62.65
SO_3_	0.00	0.00	0.00	0.23	0.00	0.00	0.00	0.27
MgO	1.43	0.88	1.42	1.03	0.00	0.00	0.00	0.55
Na_2_O	0.00	0.00	0.00	0.34	0.00	0.00	0.00	0.37
	**Aluminate (%)**				**Ferrite (%)**			
SiO_2_	4.94	5.01	6.53	4.98	3.58	4.41	5.33	3.56
Al_2_O_3_	32.02	31.30	29.28	27.57	21.70	22.90	24.66	19.07
Fe_2_O_3_	4.48	4.24	5.89	6.92	25.83	19.66	16.30	22.91
CaO	57.23	58.87	58.31	56.85	46.31	49.90	52.01	47.75
SO_3_	0.00	0.00	0.00	0.23	0.00	0.00	0.00	0.10
MgO	0.75	0.00	0.00	2.03	2.57	3.13	1.70	3.17
Na_2_O	0.58	0.59	0.00	0.53	0.00	0.00	0.00	0.31

**Table 9:** Four chemical compositions of the cement compounds proposed in this paper.

Case	Average	1	2	3	Average	1	2	3
	Alite (%)				Belite (%)			
SiO_2_	25.30	26.30	26.30	26.30	32.20	34.90	34.90	34.90
Al_2_O_3_	1.20	1.20	1.20	0.00	1.90	1.90	1.90	0.00
Fe_2_O_3_	0.70	0.70	0.70	0.00	0.90	0.90	0.90	0.00
CaO	72.70	73.70	73.70	73.70	64.80	65.10	65.10	65.10
SO_3_	0.00	0.00	0.00	0.00	0.20	0.20	0.20	0.00
	**Aluminate (%)**				**Ferrite (%)**			
SiO_2_	4.80	4.80	0.00	4.80	3.80	3.80	0.00	3.80
Al_2_O_3_	31.10	37.70	37.70	37.70	23.40	21.00	21.00	21.00
Fe_2_O_3_	6.40	6.40	0.00	6.40	22.60	32.90	32.90	32.90
CaO	57.70	62.30	62.30	62.30	50.20	46.20	46.20	46.20
SO_3_	0.00	0.00	0.00	0.00	0.00	0.00	0.00	0.00

**Table 10:** Average absolute differences between the experimental data and the results obtained with the Bogue, Taylor, and Case 1–3 models.

Compounds	Alite	Belite	Aluminate	Ferrite	Sum	Differences from the Bogue Model
Bogue	8.438	6.950	2.842	1.626	19.857	-
Taylor	5.770	6.156	2.135	2.010	16.070	3.786
Case 1	7.857	7.299	2.847	3.408	21.410	−1.554
Case 2	5.437	5.387	1.760	2.322	14.906	4.951
Case 3	5.188	5.561	4.687	2.218	17.653	2.204

**Table 11:** Average absolute differences between experimental data and the results of the 17 model sets given in Appendix B.

Phase.	Alite	Belite	Aluminate	Ferrite	Sum	Differences from the Bogue Model
Krist2	6.463	7.003	2.988	3.548	20.002	−0.146
Krist11	6.501	7.317	2.779	2.608	19.205	0.652
Krist25	6.327	7.910	3.417	5.331	22.985	−3.128
Krist27	5.865	6.167	4.916	4.994	21.942	−2.085
YamaN1	7.014	7.443	6.381	6.370	27.208	−7.351
YamaN2	6.373	6.140	3.317	2.789	18.620	1.237
YamaN3	7.805	8.244	2.816	2.463	21.328	−1.471
HarA	5.423	5.828	2.798	2.993	17.043	2.814
HarB	6.436	6.593	2.674	2.628	18.331	1.526
SRM6	11.298	9.675	2.365	2.628	25.966	−6.109
SRM7	12.854	10.755	4.745	6.291	34.646	−14.789
SRM8	6.569	6.120	2.089	2.203	16.982	2.875
CemB	8.071	6.812	2.158	3.868	20.909	−1.052
CemC	9.699	9.583	2.728	2.636	24.646	−4.789
CemD	6.012	6.220	5.341	4.810	22.383	−2.527
PAW	6.740	7.278	3.651	2.737	20.406	−0.550
Average	6.043	6.336	2.744	2.718	17.841	2.016

**Table 12:** The error percentage of the new and old models.

	Experiment (A)	Bogue (B)	Error 1 (C = A − B)	Abs. Error 1 (C’ = |A − B|)	New (D)	Error 2 (E = A − D)	Abs. Error 2 (E’ = |A − D|)
Alite (%)	61.2	53.8	−7.4	8.4	63.2	2.0	5.4
Belite (%)	17.3	22.6	5.3	6.9	16.8	−0.5	5.4
Aluminate (%)	6.5	8.7	2.1	2.8	6.8	0.3	1.8
Ferrite (%)	8.8	8.8	0.0	1.6	7.0	−1.8	2.3

**Table 13:** Applicable range of the proposed models.

	Minimum	Maximum
C/S (CaO%/SiO_2_%)	2.22(2.33) ^1^	3.21(3.30)
A/F (Al_2_O_3_%/Fe_2_O_3_%)	0.86(0.64)	-

^1^ Numbers in parentheses are the applicable range for Bogue models.

## Data Availability

The data underlying this article will be shared on reasonable request from the corresponding author.

## Appendix A

**Table A1:** Experimental Data Sets Collected from Previous Studies [15,16,17,21,22].

**Case**	**MK1**	**MK2**	**MK3**	**MK4**	**MK5**	**MK6**	**MK7**	**MK8**	**MK9**	**MK10**
CaO	64.90	65.10	64.50	62.00	68.80	64.90	64.10	65.30	64.60	65.00
SiO_2_	21.20	21.80	23.70	23.60	23.90	22.60	22.30	21.50	21.80	22.40
Al_2_O_3_	6.50	5.70	5.80	4.00	5.20	4.50	6.30	5.00	4.20	5.40
Fe_2_O_3_	3.20	3.10	1.80	2.40	2.80	1.50	2.40	2.80	4.90	2.90
SO_3_	1.00	0.60	0.60	0.30	0.10	0.60	0.40	1.10	0.40	0.30
MgO	2.40	1.30	1.20	5.00	1.90	3.60	1.60	2.20	1.60	3.20
K_2_O	1.00	0.60	0.70	0.30	0.00	0.60	0.30	0.90	0.40	0.50
Na_2_O	0.40	0.30	1.00	0.80	0.30	0.70	0.70	0.40	0.50	0.60
P2O_5_	0.10	0.10	0.10	0.10	0.20	0.20	0.10	0.10	0.10	0.10
TiO_2_	0.30	0.30	0.20	0.10	0.20	0.10	0.20	0.20	0.20	0.20
Mn_2_O_3_	0.00	0.00	0.00	0.00	0.00	0.00	0.00	0.00	0.00	0.00
LOI	0.70	1.30	0.80	0.80	0.30	0.60	1.00	1.00	1.00	0.80
Free CaO	0.50	1.00	0.80	0.50	0.10	1.10	0.30	0.50	0.20	0.30
Alite	64.20	61.30	52.60	62.80	68.50	69.60	51.50	71.40	68.60	59.50
Belite	8.70	15.60	29.80	18.20	14.50	12.50	26.60	12.00	12.50	22.10
Aluminate	8.40	8.55	10.60	5.70	2.60	7.80	11.50	2.70	0.70	6.55
Ferrite	11.25	10.55	3.60	5.80	14.35	4.35	6.20	10.20	15.70	9.10
**Case**	**MK11**	**MK12**	**MK13**	**MK14**	**MK15**	**MK16**	**MK17**	**MK18**	**MK19**	**MK20**
CaO	67.60	67.10	66.00	66.20	66.60	62.70	65.40	65.40	65.30	64.30
SiO_2_	22.40	21.90	21.50	23.00	23.20	23.70	21.00	22.60	22.60	23.60
Al_2_O_3_	6.90	4.10	5.70	4.40	5.20	5.80	6.60	4.90	4.40	5.70
Fe_2_O_3_	2.60	2.00	3.20	2.10	2.70	2.90	2.50	2.80	3.20	2.30
SO_3_	0.10	0.80	0.40	0.50	0.50	0.30	0.80	1.10	0.90	0.70
MgO	1.20	1.60	2.40	1.70	1.00	2.70	1.00	1.40	1.70	1.60
K_2_O	0.00	0.90	0.30	0.90	0.30	0.50	0.80	0.60	0.70	0.70
Na_2_O	0.30	0.40	0.80	0.50	0.30	0.70	0.40	0.40	0.50	0.60
P_2_O_5_	0.30	0.10	0.10	0.10	0.10	0.10	0.20	0.10	0.20	0.10
TiO_2_	0.20	0.20	0.20	0.20	0.20	0.20	0.20	0.20	0.20	0.20
Mn_2_O_3_	0.00	0.00	0.00	0.00	0.00	0.00	0.00	0.00	0.00	0.00
LOI	0.60	0.70	0.90	0.60	0.80	0.60	0.50	0.40	0.80	0.60
Free CaO	1.30	0.80	1.20	0.40	0.20	0.20	0.10	0.20	0.90	0.50
Alite	54.40	81.30	65.00	67.50	60.80	37.00	65.40	63.70	62.40	44.80
Belite	7.90	3.90	12.80	11.70	21.60	33.50	25.30	20.10	27.90	22.60
Aluminate	15.10	4.50	6.70	4.90	6.65	6.25	12.70	3.15	2.25	8.75
Ferrite	7.55	6.55	9.75	14.25	8.20	10.15	5.55	10.75	10.90	4.95
**Case**	**MK21**	**MK22**	**MK23**	**MK24**	**MK25**	**MK26**	**MK27**	**MK28**	**MK29**	**MK30**
CaO	65.70	64.20	65.70	66.00	66.80	69.00	66.10	67.80	66.00	68.50
SiO_2_	23.40	24.60	20.60	23.80	21.20	22.70	21.70	22.90	22.20	23.50
Al_2_O_3_	5.50	4.20	5.70	5.60	6.20	4.90	5.00	4.70	5.30	4.70
Fe_2_O_3_	1.80	2.80	4.60	3.20	4.70	1.70	4.00	2.50	2.30	1.90
SO_3_	0.40	0.60	0.40	0.70	0.10	0.10	0.20	0.20	0.30	0.10
MgO	2.10	2.10	0.80	0.80	0.90	1.30	1.80	0.80	3.30	0.80
K_2_O	0.80	0.40	0.00	0.20	0.00	0.10	0.00	0.20	0.30	0.00
Na_2_O	0.60	0.50	0.40	0.30	0.80	0.30	0.90	0.40	0.30	0.40
P_2_O_5_	0.10	0.10	0.30	0.30	0.10	0.20	0.20	0.20	0.20	0.30
TiO_2_	0.20	0.20	0.50	0.30	0.30	0.20	0.30	0.20	0.20	0.20
Mn_2_O_3_	0.00	0.00	0.00	0.00	0.00	0.00	0.00	0.00	0.00	0.00
LOI	0.50	0.40	1.20	0.90	0.60	0.70	0.90	0.60	0.40	0.60
Free CaO	0.80	0.60	1.80	0.50	1.00	1.50	2.90	1.00	0.30	0.60
Alite	68.70	54.90	55.50	49.80	59.90	72.30	68.00	69.10	69.20	72.30
Belite	14.80	31.20	22.90	32.40	16.40	12.20	10.90	16.60	9.10	14.10
Aluminate	8.90	2.25	4.80	7.35	9.05	8.70	3.45	6.85	5.85	8.50
Ferrite	4.70	10.45	16.00	10.40	12.85	4.15	13.35	6.10	11.00	3.20
**Case**	**MK31**	**MK32**	**MK33**	**MK34**	**MK35**	**MK36**	**MK37**	**MK38**	**MK39**	**CEMA**
CaO	60.20	66.20	66.40	63.60	66.40	61.90	65.30	66.70	64.90	68.70
SiO_2_	20.20	24.40	23.60	21.90	21.90	20.50	22.00	21.50	20.20	24.70
Al_2_O_3_	5.20	4.10	3.90	5.20	4.90	7.00	5.10	5.10	5.90	2.10
Fe_2_O_3_	3.90	2.90	2.70	1.80	2.40	2.60	4.00	2.40	3.50	0.40
SO_3_	0.30	0.30	0.20	1.30	0.20	1.10	0.00	0.50	0.10	1.80
MgO	6.80	1.90	1.80	3.30	1.00	3.60	1.80	1.90	0.90	0.60
K_2_O	0.50	0.10	0.00	1.00	0.30	0.70	0.00	0.60	0.30	0.06
Na_2_O	0.40	0.20	0.50	0.80	0.30	0.60	0.90	0.30	0.40	0.39
P_2_O_5_	0.40	0.10	0.20	0.20	0.20	0.20	0.10	0.20	0.30	0.45
TiO_2_	0.30	0.20	0.20	0.20	0.20	0.30	0.10	0.10	0.20	0.05
Mn_2_O_3_	0.00	0.00	0.00	0.00	0.00	0.00	0.00	0.00	0.00	0.01
LOI	1.30	0.50	0.60	0.80	1.30	0.50	1.00	0.80	0.80	1.00
Free CaO	0.20	0.10	0.20	0.90	3.60	0.80	1.10	0.80	2.10	0.00
Alite	54.60	53.60	66.10	67.50	61.30	60.20	69.90	74.30	59.80	68.90
Belite	20.60	35.20	22.90	14.90	15.30	14.10	9.20	6.20	14.90	23.40
Aluminate	5.15	0.75	2.05	8.05	6.85	8.25	4.80	4.90	7.65	3.90
Ferrite	12.30	9.10	7.90	5.15	6.35	11.50	12.10	9.45	9.45	0.00
**Case**	**CEMB**	**CEMC**	**CEMD**	**CEMF**	**CEML**	**S1**	**S2**	**S3**	**SCR**	**PAW**
CaO	61.30	64.20	63.40	63.60	66.10	64.40	66.47	63.98	63.54	65.70
SiO_2_	20.50	21.00	21.00	21.30	19.70	21.25	21.20	21.34	19.76	20.70
Al_2_O_3_	5.10	4.60	5.00	4.10	5.40	5.35	5.31	5.34	4.86	5.50
Fe_2_O_3_	3.30	2.60	2.50	5.00	3.90	4.00	4.11	4.68	2.69	2.70
SO_3_	2.80	2.80	3.00	2.10	2.10	1.54	1.69	2.54	3.75	2.60
MgO	2.80	1.80	2.10	1.60	0.90	1.27	0.94	0.88	1.45	1.50
K_2_O	1.40	0.94	1.02	0.74	0.53	0.35	0.37	0.29	0.88	0.40
Na_2_O	1.40	1.01	1.19	0.99	1.11	0.39	0.10	0.18	0.07	0.20
P_2_O_5_	0.37	0.40	0.08	0.06	0.39	0.00	0.00	0.00	0.48	0.20
TiO_2_	0.19	0.14	0.16	0.15	0.19	0.00	0.00	0.00	0.29	0.30
Mn_2_O_3_	0.05	0.03	0.04	0.04	0.04	0.00	0.00	0.00	0.03	0.00
LOI	1.90	1.30	1.30	1.00	0.30	0.00	0.00	0.00	0.00	0.00
Free CaO	0.00	0.00	0.00	0.00	0.00	0.00	0.00	0.00	0.00	0.00
Alite	49.10	62.60	66.40	58.70	66.30	58.31	59.97	50.12	69.90	66.30
Belite	24.20	16.70	14.30	17.90	14.30	19.65	19.29	21.41	8.30	8.30
Aluminate	5.70	7.40	3.70	2.30	5.10	7.76	10.07	17.27	7.50	8.70
Ferrite	11.50	7.10	9.20	16.00	14.30	8.64	8.71	6.31	6.30	7.70

## Appendix B

**Table A2:** Chemical Compositions and Calculation Models for the Cement Compound Amounts.

Set	$\mathrm{Chemical}\;\mathrm{Composition}\;a_{ij}$	Calculation Model for Cement Compounds
(1) Krist 2	$\left[\begin{array}{ccccc} 0.7215\; & 0.6498\; & 0.5654 & 0.4970\; & 0.4119\\ 0.2541 & 0.3078 & 0.0378 & 0.0314 & -\\ 0.0152\; & 0.0283\; & 0.3170 & 0.2475\; & -\\ 0.0091 & 0.0141 & 0.0798 & 0.2241 & -\\ - & - & - & - & 0.5881 \end{array}\right]$	$\left[\begin{array}{c} \mathrm{Alite}\\ \mathrm{Belite}\\ \mathrm{Aluminate}\\ \mathrm{Ferrite}\\ \mathrm{Gypsum} \end{array}\right]=\left[\begin{array}{ccccc} 5.056 & -9.920 & -7.429 & -1.617 & -3.542\\ -4.190 & 11.507 & 5.774 & 1.302 & 2.935\\ 0.118 & -0.413 & 4.216 & -4.859 & -0.083\\ 0.016 & -0.173 & -1.561 & 6.174 & -0.011\\ - & - & - & - & 1.700 \end{array}\right]\left[\begin{array}{c} \mathrm{CaO}\\ {\mathrm{SiO}}_{2}\\ {\mathrm{Al}}_{2}O_{3}\\ {\mathrm{Fe}}_{2}O_{3}\\ {\mathrm{SO}}_{3} \end{array}\right]$
(2) Krist 11	$\left[\begin{array}{ccccc} 0.7251\; & 0.6496 & 0.5931 & 0.5220\; & 0.4119\\ 0.2548\; & 0.3153 & 0.0315 & 0.0272 & -\\ 0.0141\; & 0.0250 & 0.3225 & 0.2333 & -\\ 0.0060 & 0.0100 & 0.0529 & 0.2176 & -\\ - & - & - & - & 0.5881 \end{array}\right]$	$\left[\begin{array}{c} \mathrm{Alite}\\ \mathrm{Belite}\\ \mathrm{Aluminate}\\ \mathrm{Ferrite}\\ \mathrm{Gypsum} \end{array}\right]=\left[\begin{array}{ccccc} 4.745 & -9.113 & -7.469 & -2.237 & -3.324\\ -3.844 & 10.579 & 5.750 & 1.734 & 2.692\\ 0.071 & -0.308 & 3.668 & -4.063 & -0.049\\ 0.028 & -0.159 & -0.949 & 5.566 & -0.020\\ - & - & - & - & 1.700 \end{array}\right]\left[\begin{array}{c} \mathrm{CaO}\\ {\mathrm{SiO}}_{2}\\ {\mathrm{Al}}_{2}O_{3}\\ {\mathrm{Fe}}_{2}O_{3}\\ {\mathrm{SO}}_{3} \end{array}\right]$
(3) Krist 25	$\left[\begin{array}{ccccc} 0.7211\; & 0.6529 & 0.5569 & 0.4926 & 0.4119\\ 0.2500\; & 0.3058 & 0.0449 & 0.0177 & -\\ 0.0120 & 0.0265 & 0.3323 & 0.2266 & -\\ 0.0169 & 0.0147 & 0.0658 & 0.2631 & -\\ - & - & - & - & 0.5881 \end{array}\right]$	$\left[\begin{array}{c} \mathrm{Alite}\\ \mathrm{Belite}\\ \mathrm{Aluminate}\\ \mathrm{Ferrite}\\ \mathrm{Gypsum} \end{array}\right]=\left[\begin{array}{ccccc} 5.083 & -10.131 & -6.510 & -3.227 & -3.560\\ -4.184 & 11.643 & 4.874 & 2.852 & 2.931\\ 0.259 & -0.682 & 3.322 & -3.300 & -0.181\\ -0.157 & 0.170 & -0.686 & 4.675 & 0.110\\ - & - & - & - & 1.700 \end{array}\right]\left[\begin{array}{c} \mathrm{CaO}\\ {\mathrm{SiO}}_{2}\\ {\mathrm{Al}}_{2}O_{3}\\ {\mathrm{Fe}}_{2}O_{3}\\ {\mathrm{SO}}_{3} \end{array}\right]$
(4) Krist 27	$\left[\begin{array}{ccccc} 0.7215 & 0.6500 & 0.5550 & 0.4984 & 0.4119\\ 0.2580 & 0.3235 & 0.0463 & 0.0263 & -\\ 0.0103 & 0.0153 & 0.3093 & 0.2124 & -\\ 0.0103 & 0.0112 & 0.0894 & 0.2629 & -\\ - & - & - & - & 0.5881 \end{array}\right]$	$\left[\begin{array}{c} \mathrm{Alite}\\ \mathrm{Belite}\\ \mathrm{Aluminate}\\ \mathrm{Ferrite}\\ \mathrm{Gypsum} \end{array}\right]=\left[\begin{array}{ccccc} 4.882 & -9.398 & -6.456 & -3.100 & -3.420\\ -3.899 & 10.616 & 4.665 & 2.561 & 2.731\\ 0.062 & -0.201 & 4.150 & -3.451 & -0.043\\ -0.045 & -0.017 & -1.359 & 4.990 & 0.032\\ - & - & - & - & 1.700 \end{array}\right]\left[\begin{array}{c} \mathrm{CaO}\\ {\mathrm{SiO}}_{2}\\ {\mathrm{Al}}_{2}O_{3}\\ {\mathrm{Fe}}_{2}O_{3}\\ {\mathrm{SO}}_{3} \end{array}\right]$
(5) Yama N1	$\left[\begin{array}{ccccc} 0.7359 & 0.6343 & 0.5509 & 0.4795 & 0.4119\\ 0.2443 & 0.3231 & 0.0478 & 0.0453 & -\\ 0.0131 & 0.0272 & 0.2827 & 0.2645 & -\\ 0.0067 & 0.0127 & 0.1185 & 0.2107 & -\\ - & 0.0027 & - & - & 0.5881 \end{array}\right]$	$\left[\begin{array}{c} \mathrm{Alite}\\ \mathrm{Belite}\\ \mathrm{Aluminate}\\ \mathrm{Ferrite}\\ \mathrm{Gypsum} \end{array}\right]=\left[\begin{array}{ccccc} 3.703 & -6.743 & -6.650 & 1.371 & -2.594\\ -2.814 & 8.264 & 4.530 & -1.060 & 1.971\\ 0.105 & -0.455 & 7.317 & -9.324 & -0.073\\ -0.007 & -0.028 & -4.176 & 10.008 & 0.005\\ 0.013 & -0.038 & -0.021 & 0.005 & 1.691 \end{array}\right]\left[\begin{array}{c} \mathrm{CaO}\\ {\mathrm{SiO}}_{2}\\ {\mathrm{Al}}_{2}O_{3}\\ {\mathrm{Fe}}_{2}O_{3}\\ {\mathrm{SO}}_{3} \end{array}\right]$
(6) Yama N2	$\left[\begin{array}{ccccc} 0.7264 & 0.6348 & 0.5681 & 0.4741 & 0.4119\\ 0.2555 & 0.3376 & 0.0755 & 0.0317 & -\\ 0.0119 & 0.0164 & 0.2926 & 0.2598 & -\\ 0.0062 & 0.0107 & 0.0638 & 0.2344 & -\\ - & 0.0005 & - & - & 0.5881 \end{array}\right]$	$\left[\begin{array}{c} \mathrm{Alite}\\ \mathrm{Belite}\\ \mathrm{Aluminate}\\ \mathrm{Ferrite}\\ \mathrm{Gypsum} \end{array}\right]=\left[\begin{array}{ccccc} 4.043 & -7.293 & -5.801 & -0.763 & -2.832\\ -3.057 & 8.501 & 3.485 & 1.172 & 2.141\\ -0.029 & -0.009 & 4.568 & -5.002 & 0.020\\ 0.040 & -0.192 & -1.249 & 5.595 & -0.028\\ 0.003 & -0.007 & -0.003 & -0.001 & 1.699 \end{array}\right]\left[\begin{array}{c} \mathrm{CaO}\\ {\mathrm{SiO}}_{2}\\ {\mathrm{Al}}_{2}O_{3}\\ {\mathrm{Fe}}_{2}O_{3}\\ {\mathrm{SO}}_{3} \end{array}\right]$
(7) Yama N3	$\left[\begin{array}{ccccc} 0.7370 & 0.6483 & 0.5793 & 0.4674 & 0.4119\\ 0.2442 & 0.3210 & 0.0613 & 0.0452 & -\\ 0.0122 & 0.0202 & 0.3034 & 0.2553 & -\\ 0.0062 & 0.0079 & 0.0560 & 0.2321 & -\\ 0.0005 & 0.0026 & - & - & 0.5881 \end{array}\right]$	$\left[\begin{array}{c} \mathrm{Alite}\\ \mathrm{Belite}\\ \mathrm{Aluminate}\\ \mathrm{Ferrite}\\ \mathrm{Gypsum} \end{array}\right]=\left[\begin{array}{ccccc} 4.003 & -7.680 & -6.122 & 0.165 & -2.804\\ -3.054 & 9.013 & 4.012 & -0.017 & 2.139\\ 0.056 & -0.258 & 4.081 & -4.551 & -0.039\\ -0.016 & -0.041 & -0.959 & 5.402 & 0.011\\ 0.010 & -0.034 & -0.013 & - & 1.693 \end{array}\right]\left[\begin{array}{c} \mathrm{CaO}\\ {\mathrm{SiO}}_{2}\\ {\mathrm{Al}}_{2}O_{3}\\ {\mathrm{Fe}}_{2}O_{3}\\ {\mathrm{SO}}_{3} \end{array}\right]$
(8) Har A	$\left[\begin{array}{ccccc} 0.7275 & 0.6442 & 0.5803 & 0.5097 & 0.4119\\ 0.2585 & 0.3242 & 0.0435 & 0.0409 & -\\ 0.0100 & 0.0214 & 0.3244 & 0.2376 & -\\ 0.0040 & 0.0082 & 0.0518 & 0.2108 & -\\ - & 0.0020 & - & 0.0011 & 0.5881 \end{array}\right]$	$\left[\begin{array}{c} \mathrm{Alite}\\ \mathrm{Belite}\\ \mathrm{Aluminate}\\ \mathrm{Ferrite}\\ \mathrm{Gypsum} \end{array}\right]=\left[\begin{array}{ccccc} 4.421 & -8.293 & -6.524 & -1.713 & -3.097\\ -3.540 & 9.755 & 4.831 & 1.209 & 2.479\\ 0.071 & -0.276 & 3.673 & -4.260 & -0.050\\ 0.035 & -0.152 & -0.966 & 5.778 & -0.025\\ 0.012 & -0.034 & -0.015 & -0.015 & 1.692 \end{array}\right]\left[\begin{array}{c} \mathrm{CaO}\\ {\mathrm{SiO}}_{2}\\ {\mathrm{Al}}_{2}O_{3}\\ {\mathrm{Fe}}_{2}O_{3}\\ {\mathrm{SO}}_{3} \end{array}\right]$
(9) Har B	$\left[\begin{array}{ccccc} 0.7278 & 0.6451 & 0.5867 & 0.5151 & 0.4119\\ 0.2536 & 0.3179 & 0.0535 & 0.0430 & -\\ 0.0124 & 0.0205 & 0.3009 & 0.2194 & -\\ 0.0062 & 0.0092 & 0.0589 & 0.2204 & -\\ - & 0.0072 & - & 0.0022 & 0.5881 \end{array}\right]$	$\left[\begin{array}{c} \mathrm{Alite}\\ \mathrm{Belite}\\ \mathrm{Aluminate}\\ \mathrm{Ferrite}\\ \mathrm{Gypsum} \end{array}\right]=\left[\begin{array}{ccccc} 4.467 & -8.496 & -6.814 & -1.968 & -3.129\\ -3.573 & 9.979 & 4.898 & 1.503 & 2.503\\ 0.052 & -0.249 & 4.074 & -4.127 & -0.037\\ 0.010 & -0.113 & -1.102 & 5.632 & -0.007\\ 0.044 & -0.121 & -0.056 & -0.039 & 1.670 \end{array}\right]\left[\begin{array}{c} \mathrm{CaO}\\ {\mathrm{SiO}}_{2}\\ {\mathrm{Al}}_{2}O_{3}\\ {\mathrm{Fe}}_{2}O_{3}\\ {\mathrm{SO}}_{3} \end{array}\right]$
(10) SRM 6	$\left[\begin{array}{ccccc} 0.7378 & 0.6552 & 0.5930 & 0.5157 & 0.4119\\ 0.2551 & 0.3225 & 0.0442 & 0.0430 & -\\ 0.0071 & 0.0101 & 0.3258 & 0.2138 & -\\ - & 0.0101 & 0.0370 & 0.2264 & -\\ - & 0.0020 & - & 0.0010 & 0.5881 \end{array}\right]$	$\left[\begin{array}{c} \mathrm{Alite}\\ \mathrm{Belite}\\ \mathrm{Aluminate}\\ \mathrm{Ferrite}\\ \mathrm{Gypsum} \end{array}\right]=\left[\begin{array}{ccccc} 4.425 & -8.693 & -6.630 & -2.152 & -3.099\\ -3.509 & 10.013 & 4.859 & 1.491 & 2.458\\ -0.101 & 0.193 & 3.591 & -3.197 & 0.071\\ 0.174 & -0.480 & -0.804 & 4.872 & -0.122\\ 0.012 & -0.034 & -0.015 & -0.014 & 1.692 \end{array}\right]\left[\begin{array}{c} \mathrm{CaO}\\ {\mathrm{SiO}}_{2}\\ {\mathrm{Al}}_{2}O_{3}\\ {\mathrm{Fe}}_{2}O_{3}\\ {\mathrm{SO}}_{3} \end{array}\right]$
(11) SRM 7	$\left[\begin{array}{ccccc} 0.7407 & 0.6606 & 0.5853 & 0.5307 & 0.4119\\ 0.2482 & 0.3283 & 0.0460 & 0.0530 & -\\ 0.0111 & 0.0091 & 0.2921 & 0.2341 & -\\ - & - & 0.0766 & 0.1811 & -\\ - & 0.0020 & - & 0.0011 & 0.5881 \end{array}\right]$	$\left[\begin{array}{c} \mathrm{Alite}\\ \mathrm{Belite}\\ \mathrm{Aluminate}\\ \mathrm{Ferrite}\\ \mathrm{Gypsum} \end{array}\right]=\left[\begin{array}{ccccc} 4.249 & -8.341 & -6.929 & -1.039 & -2.976\\ -3.207 & 9.351 & 4.856 & 0.372 & 2.246\\ -0.093 & 0.039 & 5.348 & -6.650 & 0.065\\ 0.039 & -0.017 & -2.262 & 8.333 & -0.028\\ 0.011 & -0.032 & -0.013 & -0.016 & 1.693 \end{array}\right]\left[\begin{array}{c} \mathrm{CaO}\\ {\mathrm{SiO}}_{2}\\ {\mathrm{Al}}_{2}O_{3}\\ {\mathrm{Fe}}_{2}O_{3}\\ {\mathrm{SO}}_{3} \end{array}\right]$
(12) SRM 8	$\left[\begin{array}{ccccc} 0.7337 & 0.6509 & 0.5686 & 0.4959 & 0.4119\\ 0.2583 & 0.3320 & 0.0240 & 0.0314 & -\\ 0.0050 & 0.0111 & 0.3493 & 0.2211 & -\\ - & - & 0.0581 & 0.2515 & -\\ 0.0030 & 0.0060 & - & - & 0.5881 \end{array}\right]$	$\left[\begin{array}{c} \mathrm{Alite}\\ \mathrm{Belite}\\ \mathrm{Aluminate}\\ \mathrm{Ferrite}\\ \mathrm{Gypsum} \end{array}\right]=\left[\begin{array}{ccccc} 4.282 & -8.140 & -6.061 & -2.099 & -2.999\\ -3.334 & 9.355 & 4.550 & 1.406 & 2.335\\ 0.052 & -0.210 & 3.285 & -2.963 & -0.036\\ -0.012 & 0.048 & -0.758 & 4.660 & 0.008\\ 0.012 & -0.054 & -0.016 & -0.004 & 1.692 \end{array}\right]\left[\begin{array}{c} \mathrm{CaO}\\ {\mathrm{SiO}}_{2}\\ {\mathrm{Al}}_{2}O_{3}\\ {\mathrm{Fe}}_{2}O_{3}\\ {\mathrm{SO}}_{3} \end{array}\right]$
(13) Cem B	$\left[\begin{array}{ccccc} 0.7144 & 0.6445 & 0.5800 & 0.4753 & 0.4119\\ 0.2507 & 0.3209 & 0.0501 & 0.0368 & -\\ 0.0206 & 0.0207 & 0.3245 & 0.2228 & -\\ 0.0143 & 0.0139 & 0.0454 & 0.2651 & -\\ - & - & - & - & 0.5881 \end{array}\right]$	$\left[\begin{array}{c} \mathrm{Alite}\\ \mathrm{Belite}\\ \mathrm{Aluminate}\\ \mathrm{Ferrite}\\ \mathrm{Gypsum} \end{array}\right]=\left[\begin{array}{ccccc} 4.915 & -9.346 & -7.129 & -1.526 & -3.442\\ -3.829 & 10.429 & 5.072 & 1.157 & 2.682\\ -0.026 & -0.050 & 3.545 & -2.925 & 0.018\\ -0.060 & -0.033 & -0.487 & 4.294 & 0.042\\ - & - & - & - & 1.700 \end{array}\right]\left[\begin{array}{c} \mathrm{CaO}\\ {\mathrm{SiO}}_{2}\\ {\mathrm{Al}}_{2}O_{3}\\ {\mathrm{Fe}}_{2}O_{3}\\ {\mathrm{SO}}_{3} \end{array}\right]$
(14) Cem C	$\left[\begin{array}{ccccc} 0.7279 & 0.6621 & 0.5922 & 0.5151 & 0.4119\\ 0.2467 & 0.3228 & 0.0504 & 0.0455 & -\\ 0.0113 & 0.0150 & 0.3148 & 0.2364 & -\\ 0.0141 & - & 0.0426 & 0.2029 & -\\ - & - & - & - & 0.5881 \end{array}\right]$	$\left[\begin{array}{c} \mathrm{Alite}\\ \mathrm{Belite}\\ \mathrm{Aluminate}\\ \mathrm{Ferrite}\\ \mathrm{Gypsum} \end{array}\right]=\left[\begin{array}{ccccc} 4.594 & -9.908 & -6.984 & -1.483 & -3.218\\ -3.503 & 10.057 & 4.844 & 0.990 & 2.453\\ 0.288 & -0.748 & 3.360 & -4.478 & -0.202\\ -0.380 & 0.789 & -0.220 & 5.971 & 0.266\\ - & - & - & - & 1.700 \end{array}\right]\left[\begin{array}{c} \mathrm{CaO}\\ {\mathrm{SiO}}_{2}\\ {\mathrm{Al}}_{2}O_{3}\\ {\mathrm{Fe}}_{2}O_{3}\\ {\mathrm{SO}}_{3} \end{array}\right]$
(15) Cem D	$\left[\begin{array}{ccccc} 0.7222 & 0.6498 & 0.5831 & 0.5291 & 0.4119\\ 0.2490 & 0.3200 & 0.0653 & 0.0542 & -\\ 0.0182 & 0.0209 & 0.2928 & 0.2509 & -\\ 0.0107 & 0.0093 & 0.0589 & 0.1658 & -\\ - & - & - & - & 0.5881 \end{array}\right]$	$\left[\begin{array}{c} \mathrm{Alite}\\ \mathrm{Belite}\\ \mathrm{Aluminate}\\ \mathrm{Ferrite}\\ \mathrm{Gypsum} \end{array}\right]=\left[\begin{array}{ccccc} 4.702 & -9.048 & -7.081 & -1.331 & -3.294\\ -3.653 & 10.200 & 4.785 & 1.081 & 2.559\\ 0.074 & -0.248 & 4.821 & -7.450 & -0.052\\ -0.123 & 0.096 & -1.525 & 8.701 & 0.086\\ - & - & - & - & 1.700 \end{array}\right]\left[\begin{array}{c} \mathrm{CaO}\\ {\mathrm{SiO}}_{2}\\ {\mathrm{Al}}_{2}O_{3}\\ {\mathrm{Fe}}_{2}O_{3}\\ {\mathrm{SO}}_{3} \end{array}\right]$
(16) Paw	$\left[\begin{array}{ccccc} 0.7176 & 0.6392 & 0.5888 & 0.5113 & 0.4119\\ 0.2620 & 0.3352 & 0.0516 & 0.0381 & -\\ 0.0123 & 0.0178 & 0.2856 & 0.2042 & -\\ 0.0057 & 0.0051 & 0.0717 & 0.2453 & -\\ 0.0023 & 0.0028 & 0.0024 & 0.0011 & 0.5881 \end{array}\right]$	$\left[\begin{array}{c} \mathrm{Alite}\\ \mathrm{Belite}\\ \mathrm{Aluminate}\\ \mathrm{Ferrite}\\ \mathrm{Gypsum} \end{array}\right]=\left[\begin{array}{ccccc} 4.558 & -8.224 & -7.361 & -2.077 & -3.192\\ -3.567 & 9.464 & 5.221 & 1.606 & 2.498\\ 0.060 & -0.289 & 4.362 & -3.711 & -0.042\\ -0.050 & 0.080 & -1.212 & 5.176 & 0.035\\ -0.002 & -0.010 & -0.011 & 0.006 & 1.702 \end{array}\right]\left[\begin{array}{c} \mathrm{CaO}\\ {\mathrm{SiO}}_{2}\\ {\mathrm{Al}}_{2}O_{3}\\ {\mathrm{Fe}}_{2}O_{3}\\ {\mathrm{SO}}_{3} \end{array}\right]$
(17) Case Avg	$\left[\begin{array}{ccccc} 0.7274 & 0.6483 & 0.5768 & 0.5017 & 0.4119\\ 0.2527 & 0.3223 & 0.0483 & 0.0381 & -\\ 0.0123 & 0.0191 & 0.3108 & 0.2337 & -\\ 0.0073 & 0.0086 & 0.0641 & 0.2260 & -\\ 0.0004 & 0.0017 & 0.0001 & 0.0004 & 0.5881 \end{array}\right]$	$\left[\begin{array}{c} \mathrm{Alite}\\ \mathrm{Belite}\\ \mathrm{Aluminate}\\ \mathrm{Ferrite}\\ \mathrm{Gypsum} \end{array}\right]=\left[\begin{array}{ccccc} 4.471 & -8.538 & -6.637 & -1.617 & -3.131\\ -3.510 & 9.836 & 4.731 & 1.238 & 2.459\\ 0.060 & -0.245 & 4.021 & -4.249 & -0.042\\ -0.028 & -0.029 & -1.105 & 5.633 & 0.019\\ 0.008 & -0.024 & -0.010 & -0.005 & 1.695 \end{array}\right]\left[\begin{array}{c} \mathrm{CaO}\\ {\mathrm{SiO}}_{2}\\ {\mathrm{Al}}_{2}O_{3}\\ {\mathrm{Fe}}_{2}O_{3}\\ {\mathrm{SO}}_{3} \end{array}\right]$
(18) Case 1	$\left[\begin{array}{ccccc} 0.7224 & 0.6325 & 0.5597 & 0.4445 & 0.4119\\ 0.2580 & 0.3389 & 0.0434 & 0.0367 & -\\ 0.0121 & 0.0186 & 0.3392 & 0.2020 & -\\ 0.0071 & 0.0083 & 0.0576 & 0.3164 & -\\ 0.0004 & 0.0017 & 0.0001 & 0.0004 & 0.5881 \end{array}\right]$	$\left[\begin{array}{c} \mathrm{Alite}\\ \mathrm{Belite}\\ \mathrm{Aluminate}\\ \mathrm{Ferrite}\\ \mathrm{Gypsum} \end{array}\right]=\left[\begin{array}{ccccc} 4.098 & -7.295 & -5.601 & -1.331 & -2.870\\ -3.123 & 8.533 & 3.907 & 0.900 & 2.187\\ 0.035 & -0.193 & 3.274 & -2.118 & -0.025\\ -0.017 & -0.025 & -0.572 & 3.552 & 0.012\\ 0.006 & -0.020 & -0.008 & -0.004 & 1.696 \end{array}\right]\left[\begin{array}{c} \mathrm{CaO}\\ {\mathrm{SiO}}_{2}\\ {\mathrm{Al}}_{2}O_{3}\\ {\mathrm{Fe}}_{2}O_{3}\\ {\mathrm{SO}}_{3} \end{array}\right]$
(19) Case 2	$\left[\begin{array}{ccccc} 0.7224 & 0.6325 & 0.6226 & 0.4616 & 0.4119\\ 0.2580 & 0.3389 & - & - & -\\ 0.0121 & 0.0186 & 0.3774 & 0.2198 & -\\ 0.0071 & 0.0083 & - & 0.3286 & -\\ 0.0004 & 0.0017 & - & - & 0.5881 \end{array}\right]$	$\left[\begin{array}{c} \mathrm{Alite}\\ \mathrm{Belite}\\ \mathrm{Aluminate}\\ \mathrm{Ferrite}\\ \mathrm{Gypsum} \end{array}\right]=\left[\begin{array}{ccccc} 4.088 & -7.212 & -6.745 & -1.436 & -2.863\\ -3.113 & 8.442 & 5.136 & 1.093 & 2.180\\ 0.028 & -0.153 & 2.604 & -1.702 & -0.020\\ -0.010 & -0.058 & 0.016 & 3.047 & 0.007\\ 0.006 & -0.020 & -0.011 & -0.002 & 1.696 \end{array}\right]\left[\begin{array}{c} \mathrm{CaO}\\ {\mathrm{SiO}}_{2}\\ {\mathrm{Al}}_{2}O_{3}\\ {\mathrm{Fe}}_{2}O_{3}\\ {\mathrm{SO}}_{3} \end{array}\right]$
(20) Case 3	$\left[\begin{array}{ccccc} 0.7368 & 0.6512 & 0.5597 & 0.4445 & 0.4119\\ 0.2632 & 0.3488 & 0.0434 & 0.0367 & -\\ - & - & 0.3392 & 0.2020 & -\\ - & - & 0.0576 & 0.3164 & -\\ - & - & 0.0001 & 0.0004 & 0.5881 \end{array}\right]$	$\left[\begin{array}{c} \mathrm{Alite}\\ \mathrm{Belite}\\ \mathrm{Aluminate}\\ \mathrm{Ferrite}\\ \mathrm{Gypsum} \end{array}\right]=\left[\begin{array}{ccccc} 4.071 & -7.600 & -5.522 & -1.309 & -2.852\\ -3.071 & 8.600 & 3.818 & 0.878 & 2.151\\ - & - & 3.306 & -2.111 & -\\ - & - & -0.602 & 3.544 & -\\ - & - & - & -0.002 & 1.700 \end{array}\right]\left[\begin{array}{c} \mathrm{CaO}\\ {\mathrm{SiO}}_{2}\\ {\mathrm{Al}}_{2}O_{3}\\ {\mathrm{Fe}}_{2}O_{3}\\ {\mathrm{SO}}_{3} \end{array}\right]$
